# Coupled Effect of Interfacial Grit Particles and TGO Amplitude on Bond-Coat Crack Propagation in Thermal Barrier Coatings

**DOI:** 10.3390/ma19102025

**Published:** 2026-05-13

**Authors:** Jianping Lai, Xin Shen, Xiaohu Yuan, Zhiming Gao, Xiufang Gong, Yuhang Zhang, Mengli Liu, Jiaxin Yu, Qiyuan Li, Zhiyuan Wei, Bingbing Liu

**Affiliations:** 1 Department of Materials Science and Engineering, Tianjin University, Tianjin 300047, China; ljp@swust.edu.cn; 2 Key Laboratory of Testing Technology for Manufacturing Process in Ministry of Education, State Key Laboratory of Environment-Friendly Energy Materials, Southwest University of Science and Technology, Mianyang 621010, China; 3 State Key Laboratory of Clean and Efficient Turbomachinery Power Equipment, Deyang 618000, China; 4 School of Automotive Materials, Hubei University of Automotive Technology, Shiyan 442002, China; 20240044@huat.edu.cn (Z.W.); l18062304823@163.com (B.L.)

**Keywords:** thermal barrier coatings, residual grit particles, thermally grown oxide, crack propagation, finite element modeling

## Abstract

Residual grit particles introduced during grit blasting are important process-induced defects that can significantly affect the interfacial damage evolution of thermal barrier coatings (TBCs) under thermal cycling; however, the coupled effects of thermally grown oxide (TGO) amplitude, grit size, and grit position on crack propagation in the bond coat (BC) remain insufficiently understood. In this work, a two-dimensional finite element model containing residual alumina grit particles was established to investigate the influence of these three factors on the radial stress distribution and crack growth behavior in the BC, and their individual contributions and interaction effects were further quantified using response surface methodology. The results showed that TGO morphology and interfacial grit defects jointly controlled the stress concentration and crack propagation behavior in the BC. Increasing the TGO amplitude intensified the radial tensile stress concentration in the BC and gradually shifted the critical stress region during thermal cycling. Larger grit particles further aggravated the local stress concentration near the grit tips, while the movement of grit particles toward the TGO peak led to a more pronounced increase in stress concentration and crack propagation tendency. The crack growth behavior was found to be consistent with the corresponding stress evolution characteristics. Response surface analysis further revealed that grit size and grit position had much stronger effects on crack propagation than TGO amplitude, and their interaction was the most significant among all factor combinations. The minimum crack length in the BC layer was obtained at a TGO amplitude of 0.01 mm, a grit size of 20 μm, and a position parameter of 0.752, and the predicted value agreed well with the finite element result. This study provides a comparative basis for interfacial damage assessment and grit-blasting parameter optimization in TBCs containing residual grit defects.

## 1. Introduction

Thermal barrier coatings (TBCs), as an important surface protection system for high-temperature components in gas turbines and aero-engines, generally consist of a ceramic top coat (TC), a thermally grown oxide (TGO) layer, a metallic bond coat (BC), and a superalloy substrate (SUB) [1,2]. The TGO layer is typically composed of α-Al_2_O_3_, and its formation and growth are mainly associated with the inward diffusion of oxygen through the TC and the selective oxidation of Al in the BC during service [3]. As a key transition layer in the TBC system, the TGO can, to some extent, inhibit further oxidation and improve the high-temperature stability of the coating system [4]. However, under thermal cycling conditions, the continuous growth of the TGO and the accumulation of residual stresses caused by the thermal expansion mismatch between adjacent layers often become major factors leading to coating failure [5,6]. Ranjbar et al. [7] employed the Debond technique to investigate the effects of TGO geometry and amplitude on stress distribution and interfacial crack propagation, while Zhang et al. [8] used the extended finite element method to study the influence of TGO amplitude on crack propagation behavior in the TC. In addition to interfacial morphology, the dynamic growth behavior of the TGO also plays a significant role in coating damage evolution. Wei et al. [9] considered the dynamic growth of the TGO and found that TGO growth markedly increased the probability of crack initiation in the ceramic top coat. Zhang et al. [10] established a TGO growth control equation based on oxygen diffusion distribution and demonstrated that non-uniform TGO growth significantly affected the initiation time of internal cracks in the coating. Hu et al. [11] investigated the effects of TGO composition and the location of internal oxides on the interfacial stress distribution.

However, existing studies have mainly focused on the influence of TGO morphological evolution and growth behavior on coating failure, while insufficient attention has been paid to practical processing-related factors. In fact, during TBC fabrication, grit blasting is commonly applied to the substrate surface to increase roughness and enhance coating adhesion [12,13], but this process often leads to partial retention of grit particles on the substrate surface [14]. Giouse et al. [15] reported that residual grit particles can alter the local microstructure and mechanical properties near the substrate surface and may promote preferential crack initiation around the embedded particles. Antoš et al. [16] further pointed out that residual grit particles remaining on the substrate surface can act as major stress concentration sites within the coating under high-temperature cyclic loading. These findings indicate that residual grit particles introduced by grit blasting may interact with TGO morphological evolution and growth behavior, thereby further affecting the internal stress distribution and crack propagation process in TBCs. Nevertheless, the underlying coupling mechanism has not yet been systematically clarified.

Therefore, in this study, a TBC system containing residual grit particles was selected as the research object, and a two-dimensional thermo-mechanically coupled finite element model considering both TGO undulation morphology and grit inclusions was established. A UEXPAN subroutine was employed to simulate the anisotropic equivalent growth of the TGO, and the crack propagation behavior within the BC during thermal cycling was analyzed using XFEM. On this basis, the TGO amplitude, grit size, and grit position were selected as variables, and the response surface methodology was further adopted to quantitatively evaluate the relative contributions of these factors to the crack propagation length within the BC.

## 2. Model Description

### 2.1. Geometry Model

**Figure 1:**
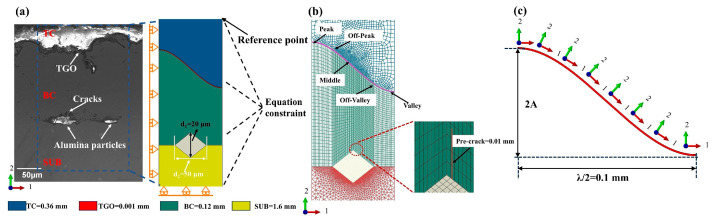
Geometry and mesh of the thermal barrier coating system: (**a**) geometrical model and boundary conditions; (**b**) mesh details; (**c**) TGO growth directions.

Using Abaqus 2022 [17], a typical two-dimensional finite element model of the TBCs was established [18,19]. From top to bottom, the coating system consisted of a TC layer with a thickness of 0.36 mm, a TGO layer with a thickness of 0.001 mm, a BC layer with a thickness of 0.12 mm, and a SUB with a thickness of 1.6 mm. To account for the influence of residual grit particles on crack distribution during thermal cycling, grit inclusions were introduced at the interface between the substrate and the bond coat using the partition tool in Abaqus. As observed from the cross-sectional scanning electron microscopy (SEM) image of the TBCs shown in Figure 1, the residual grit particles exhibited an irregular flattened morphology, while the TGO interface showed a complex undulating profile. In addition, irregular cracks were found to extend around the grit particles and the TGO layer after thermal shock.

**Figure 2:**
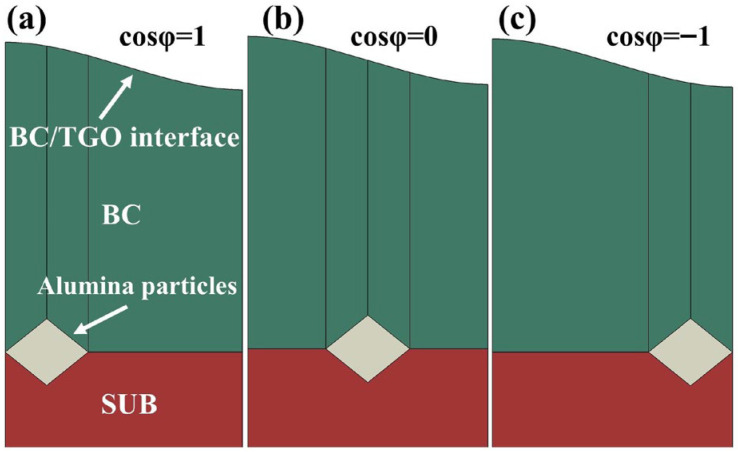
Schematic geometrical models of different grit positions: (**a**) cosφ = 1; (**b**) cosφ = 0; (**c**) cosφ = −1.

To improve computational efficiency, the geometrical features of both the grit particles and the TGO interface were simplified in the present simulation. Such a two-dimensional representation has been widely used as a reasonable and computationally efficient framework for investigating TGO morphology and coating defects in TBC systems [20,21,22]. The grit particle was modeled as a diamond-shaped quadrilateral with diagonal lengths of d_2_ = 50 μm and d_1_ = 20 μm. This simplified geometry was selected based on the cross-sectional SEM morphology shown in Figure 1a and was intended to retain the main geometrical characteristics relevant to local stress concentration and crack propagation. Since residual grit particles in actual grit-blasted interfaces are not embedded at a fixed position but instead exhibit different locations relative to the TGO peaks and valleys, a position parameter, cosφ, was introduced to characterize the variation in grit location. The corresponding geometric model is shown in Figure 2. The TGO interface was represented by a classical cosine function [23,24,25].

### 2.2. Boundary Condition and Meshing

To evaluate the effect of stress concentration near the grit tip on crack propagation, an initial vertical crack with a length of 0.01 mm was prefabricated in the BC layer near the upper tip of the grit particle, serving as the crack starter for subsequent crack propagation analysis. Considering the periodic nature of the interfacial morphology in TBCs, one interface period was selected as the computational domain to improve computational efficiency, and periodic boundary conditions were applied to both lateral sides of the model. For this model, a symmetry constraint was imposed on the left boundary, such that the displacement of all nodes along the left boundary was constrained in the 1-direction. On the right boundary, an equation constraint was applied so that all nodes on the right boundary had the same 1-direction displacement as the reference point. The displacement of the bottom boundary was constrained in the 2-direction to prevent rigid-body motion, while all degrees of freedom were released on the top boundary. The detailed geometric parameters and boundary conditions are shown in Figure 1.

A transient sequentially coupled thermo-mechanical analysis was employed to capture the temperature evolution during thermal cycling and the associated thermal mismatch stresses. A transient heat transfer step was first performed to obtain the temperature field at each instant, and the resulting temperature field was then imported into the static analysis as a predefined field for stress–strain calculation. In the thermal analysis, four-node linear heat transfer quadrilateral elements (DC2D4) were used throughout the model. In the mechanical analysis, four-node plane strain quadrilateral elements (CPE4) were assigned to the TC, TGO, BC, and grit particle, whereas the substrate was meshed with three-node plane strain triangular elements (CPE3). To account for the anisotropic growth of the TGO layer, a swept meshing strategy was adopted for the TGO region.

### 2.3. Thermal Cycling History

**Figure 3:**
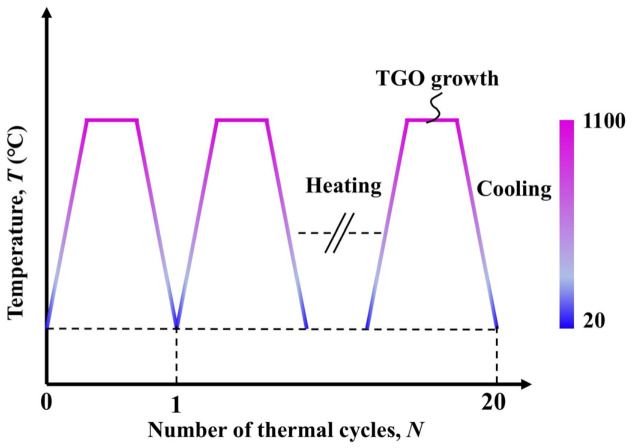
Thermal loading history.

To investigate the stress evolution of TBCs under cyclic thermal loading and the effect of grit size on crack propagation behavior, cyclic thermal loads were applied in the numerical simulations. In each thermal cycle, the specimen was first heated from 20 °C to 1100 °C over 300 s, held at 1100 °C for 600 s, and then cooled to 20 °C over 300 s. To reduce the computational cost, 20 thermal cycles were considered. The applied cyclic thermal loading profile is shown in Figure 3. A uniform temperature field was imposed on the entire model, and no temperature gradient was assumed to exist among the coating layers during the thermal cycling process [26].

### 2.4. Material Property

Except for the TGO layer, all other constituents in the model were assumed to be isotropic and spatially homogeneous. The BC layer was modeled as an elastic–plastic material, and its plastic parameters are listed in Table 1 [27,28]. The TC, SUB, and residual grit particles were treated as linear elastic materials, since creep and plastic deformation of the substrate have a negligible effect on the stress distribution in TBCs. The initial residual stresses introduced during coating deposition, as well as high-temperature phase transformation and creep during service, were not considered in the present model. This simplification may affect the quantitative accuracy of the stress and crack length; therefore, the present model is mainly intended to reveal relative trends and coupling effects. Because TBCs are subjected to significant temperature variations during service, all thermophysical and mechanical properties were defined as temperature-dependent to ensure the accuracy of the simulation. The temperature-dependent material properties of each constituent in the TBC system are summarized in Table 2 [29,30].

**Figure 4:**
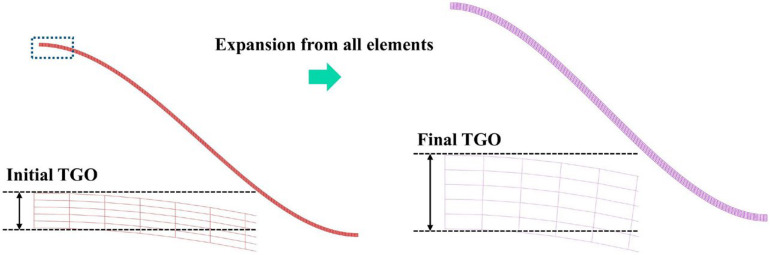
TGO growth modes.

Considering that the TGO layer continuously thickens during thermal cycling, the volume expansion and strain accumulation induced by its growth can significantly alter the interfacial stress state of the coating system and increase the tendency for crack propagation [31]. To incorporate this effect into the finite element model, the Abaqus user subroutine UEXPAN was employed to impose a time-dependent equivalent expansion strain during the high-temperature dwelling stage. The equivalent thickening of the TGO was achieved by applying element expansion to the initial TGO elements, thereby simulating its anisotropic growth behavior in an equivalent manner, as shown in Figure 4.

In Abaqus, the UEXPAN subroutine was used to simulate TGO growth by prescribing an equivalent expansion strain $\varepsilon^{\exp}$. The equivalent expansion strain of the TGO was decomposed into two components: an irreversible growth strain $\varepsilon^{g}$, associated with time and temperature, and a reversible thermal strain $\varepsilon^{th}$.

(1) $$\varepsilon^{\exp}(t,T)=\varepsilon^{g}(t)+\varepsilon^{th}(T)$$

where *t* denotes time, and *T* denotes temperature. The thermal expansion strain was defined using a linear thermal expansion relationship:

(2) $$\varepsilon^{th}(T)=\alpha_{TGO}(T-T_{ref})I$$

where $\alpha_{TGO}$ is the thermal expansion coefficient of TGO, taken as 9.8 × 10^−6^∙°C^−1^; $T_{ref}$ is the reference temperature, set to 20 °C; and *I* is the identity tensor.

Because TGO growth is strongly directional, the growth strain was divided into an in-plane direction (tangential, denoted as 1) and a through-thickness direction (normal, denoted as 2). The ratio between these two components was set to 0.1 to reflect the preferential growth of TGO in the thickness direction: $\frac{\varepsilon_{1}^{g}}{\varepsilon_{2}^{g}}=0.1$ [31].

**Figure 5:**
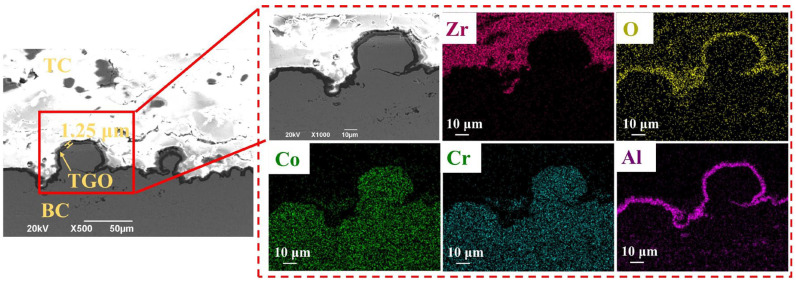
Cross-sectional SEM image of the TBC interface after 20 thermal cycles and corresponding elemental distribution maps.

The initial geometric thickness of the TGO in the model was set to 1 μm. According to the cross-sectional SEM observation of the TGO layer after 20 thermal cycles, the measured TGO thickness was approximately 1.25 μm, as illustrated in Figure 5; therefore, the total equivalent growth increment in the thickness direction after 20 cycles was $\Delta h_{2,tot}=2.5\times 10^{-4}$ mm. Based on the prescribed ratio of $\frac{\varepsilon_{1}^{g}}{\varepsilon_{2}^{g}}=0.1$, the corresponding total equivalent growth increment in the in-plane direction was $\Delta h_{1,tot}=2.5\times 10^{-5}$. Taking the initial TGO thickness $h_{0}=1$ μm as the reference, the total growth strains in the thickness and in-plane directions after 20 thermal cycles were obtained as $\varepsilon_{2,tot}^{g}=\frac{\Delta h_{2,tot}}{h_{0}}=0.25$, $\varepsilon_{1,tot}^{g}=\frac{\Delta h_{1,tot}}{h_{0}}=0.025$.

Considering that TGO growth mainly occurs during the high-temperature holding stage, a time-window criterion was introduced in UEXPAN to identify the active growth period within each thermal cycle. The duration of a single thermal cycle was as follows:


(3) $$T_{\mathrm{cycle}}=t_{heat}+t_{hold}+t_{cool}=300+600+300=1200\;s$$

The subroutine first reads the global accumulated time provided by Abaqus, *t* = TIME (2), and then determines the time position within the current cycle through a modulo operation:


(4) $$t_{in}=\mathrm{mod}(t,T_{cycle})\in \left[0,1200\right)$$

When $t_{in}\in \left[300,900\right]$ s, the model is considered to be in the high-temperature holding stage. During this period, the irreversible growth strain is accumulated incrementally according to the prescribed growth rate and time increment. During the heating and cooling stages, growth-strain accumulation is suspended, and only the thermal expansion strain is retained. To ensure continuous accumulation of growth strain across thermal cycles, the growth strain was stored in the state variable STATEV and output through the field variable SDV. Specifically, SDV1 = STATEV (1) represents the accumulated growth strain $\varepsilon_{1}^{g}$ in direction 1, while SDV2 = STATEV (2) represents the accumulated growth strain $\varepsilon_{2}^{g}$ in direction 2 (through-thickness direction). For each increment satisfying the holding-stage criterion, the growth strain in direction 1 was updated as follows:

(5) $$\varepsilon_{1}^{g}(t\;+\;\Delta t)=\varepsilon_{1}^{g}(t)+{\overset{•}{\varepsilon }}_{1}^{g}\Delta t$$

where $\varepsilon_{1}^{g}(t)$ denotes the accumulated growth strain in direction 1 at time *t*, which is a history-dependent variable that increases monotonically with time, and $\varepsilon_{1}^{g}(t+\Delta t)$ is the updated value after the current increment; ${\overset{•}{\varepsilon }}_{1}^{g}$ is the growth-strain rate in direction 1. Assuming that the total growth strain is uniformly distributed over the entire holding period, this rate was obtained by dividing the total growth strain in direction 1 by the total holding time. The growth strain in direction 2 was calculated in the same manner.

**Figure 6:**
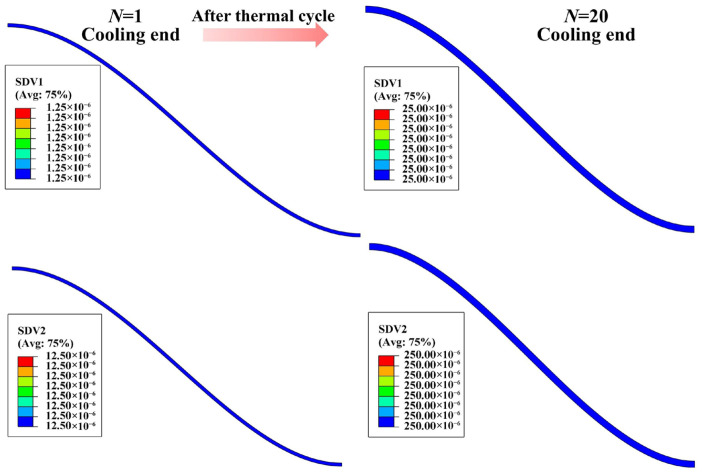
Evolution of TGO growth-strain state variables (SDVs) with thermal cycle number at the end of each thermal cycle.

As thermal cycling proceeds, the SDV field provides a direct visualization of the spatial distribution of growth strain in different directions and can be used to verify whether the strain accumulates continuously and correctly with increasing cycle number, as illustrated in Figure 6. Here, SDV1 represents the accumulated growth strain of the TGO in the in-plane direction, whereas SDV2 represents that in the thickness direction. As the number of thermal cycles increases from 1 to 20, both SDV1 and SDV2 gradually increase, indicating that the developed subroutine successfully achieves continuous accumulation of growth strain during cyclic loading. Meanwhile, SDV2 remains significantly larger than SDV1 throughout the cycling process, with the ratio SDV1/SDV2 remaining approximately 0.1, confirming that the prescribed anisotropic TGO growth mode is correctly implemented.

### 2.5. Crack Initiation and Growth Criterion

Crack propagation along arbitrary paths within the coating was simulated using the extended finite element method (XFEM) [32]. In conventional finite element analysis, modeling crack growth is cumbersome because the mesh must be continuously updated to accommodate geometric discontinuities. The XFEM proposed by Belytschko and Black introduces additional degrees of freedom and enrichment functions into the conventional finite element framework based on the partition of unity concept, thereby effectively overcoming the limitations associated with crack propagation along arbitrary paths and displacement discontinuities [33].

In this study, XFEM based on a linear traction–separation law was employed to simulate arbitrary crack propagation within the BC layer during thermal cycling. First, the maximum principal stress criterion was adopted as the damage initiation criterion to describe crack propagation within the BC layer:

(6) $$\frac{\langle \sigma_{\max}\rangle }{\sigma_{\max}^{c}}=1$$

where $\sigma_{\max}^{c}$ is the critical maximum principal stress, and the maximum principal stress for crack initiation in the BC layer was taken as 600 MPa [34]. Subsequently, an energy-based power-law criterion was adopted to describe mixed-mode crack propagation inside the BC layer:

(7) $${\left\{\frac{G_{n}}{G_{n}^{C}}\right\}}^{\alpha_{n}}+{\left\{\frac{G_{s}}{G_{s}^{c}}\right\}}^{\alpha_{s}}=1$$

where $G_{n}^{c}$ and $G_{s}^{c}$ are the critical fracture energies required for failure in the normal and first shear directions, respectively. The exponent was set to 1, and the fracture energy components in all directions were assumed to be equal, with a value of 50 J/m^2^ [35].

### 2.6. Model Reliability Validation

**Figure 7:**
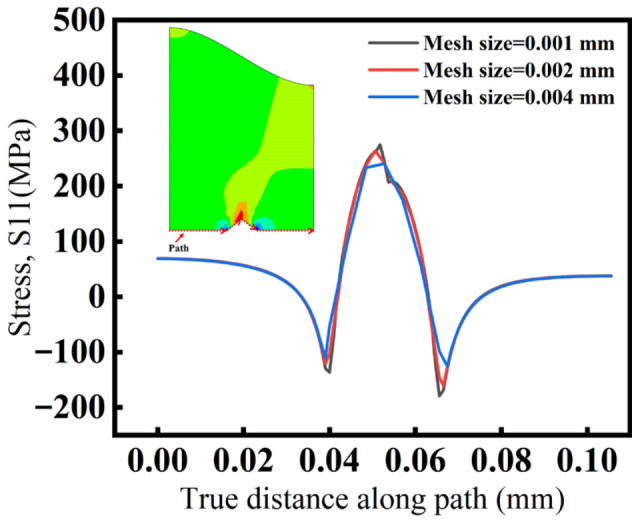
Mesh-independence verification.

First, the mesh size near the grit tip was gradually refined from 0.004 mm to 0.001 mm. The S11 stress distribution along the same extraction path at the bottom of the BC layer was then compared to ensure that the numerical results were not affected by the mesh size and to verify that no stress singularity existed near the grit tip in the BC layer. As shown in Figure 7, with mesh refinement, the radial tensile stress (S11) curves overlap well over most of the path, and only slight differences appear near the grit tip, which gradually diminish as the mesh is further refined. This indicates that the numerical results are insensitive to mesh size and that the numerical solution satisfies the mesh convergence requirement. Moreover, the S11 stress at the grit tip in the BC layer tends to stabilize with mesh refinement, indicating that the stress concentration at the grit tip is a stress concentration effect rather than a true stress singularity. Considering the computational accuracy required for subsequent crack propagation analysis, a local mesh size of 0.001 mm was adopted near the grit tip in the BC layer.

## 3. Results and Discussion

### 3.1. Stress Distribution Inside the BC Layer Under Single-Factor Conditions

Under high-temperature thermal cycling, the gas-turbine thermal barrier coating develops significant thermal mismatch and stress concentration owing to the mismatch in the coefficients of thermal expansion among different layers and the interfacial defects introduced during spraying. These stresses provide the main driving force for crack propagation. At present, opening-mode (Mode I) cracks are the most common and most dangerous crack type in TBCs. The load acting on Mode I cracks is mainly tensile stress perpendicular to the crack surface, under which the crack gradually propagates. The stress evolution inside the coating during thermal cycling affects the distribution of critical sites for crack nucleation, and therefore, the analysis of the internal stress evolution is of great importance. Radial tensile stress (S11) and normal tensile stress (S22) are the main factors leading to Mode I fracture [36]. Positive S22 mainly induces the propagation of horizontal cracks, whereas positive S11 leads to the propagation of vertical cracks [37]. Because the predefined crack in the BC layer is approximately vertical in the present model, S11 is used here as the relevant stress indicator for evaluating its opening tendency and propagation behavior. Therefore, this section focuses on the distribution and evolution of S11 in the BC layer under single-factor conditions, with particular attention to the effects of TGO amplitude, grit size, and grit position during thermal cycling.

**Figure 8:**
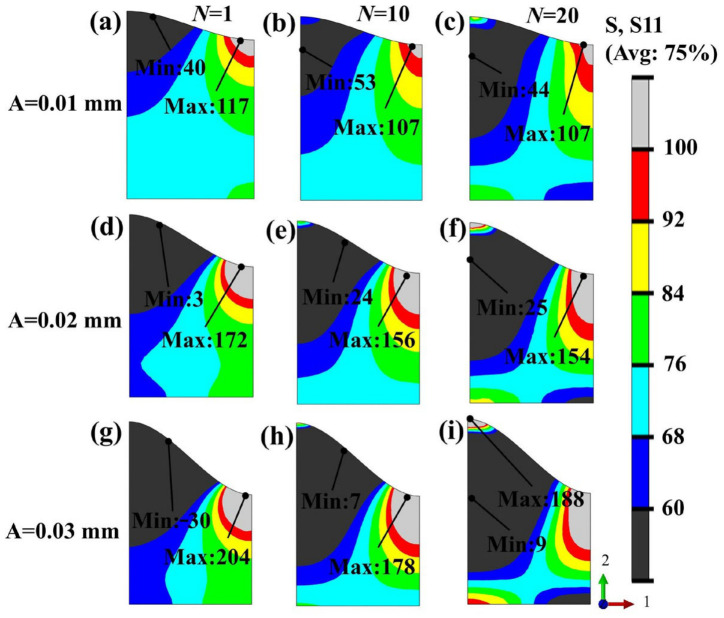
Distribution of radial stress (S11) on the BC layer surface under different TGO amplitudes during thermal cycling: (**a**–**c**) A = 0.01 mm; (**d**–**f**) A = 0.02 mm; (**g**–**i**) A = 0.03 mm.

Cracking in thermal barrier coatings generally occurs during cooling [7,38]. Therefore, the present study mainly analyzes the evolution of stress distribution at the end of cooling after multiple thermal cycles. As shown in Figure 8, in the absence of grit inclusions, the TGO amplitude has a significant effect on the stress distribution in the BC layer. When the TGO amplitude is A = 0.01 mm, the S11 stress in the BC layer is dominated overall by tensile stress. In the first cycle, the maximum and minimum values are 117 MPa and 40 MPa, respectively. By the 10th and 20th cycles, the maximum values both decrease to 107 MPa, while the minimum values are 53 MPa and 44 MPa, respectively. The maximum stress is mainly located near the wave valley, whereas the minimum stress is located near the wave peak. As thermal cycling proceeds, plastic strain gradually accumulates in the BC layer and produces a certain stress-relaxation effect on the local stress concentration [39], resulting in a slight decrease in the overall stress level. The case of A = 0.02 mm exhibits a similar evolution trend. When the TGO amplitude is further increased to 0.03 mm, stress concentration inside the BC layer becomes more pronounced. In the first cycle, the maximum S11 reaches 204 MPa, while the minimum value drops to −30 MPa, indicating that obvious compressive stress has already appeared in local regions due to the increased TGO amplitude. In the 10th cycle, the maximum and minimum values are 178 MPa and 7 MPa, respectively; by the 20th cycle, the maximum rebounds to 188 MPa, and the minimum becomes 9 MPa. Compared with the other two amplitudes, the large-amplitude condition not only leads to higher stress peaks but also causes the location of the maximum stress to shift from the vicinity of the wave valley to the wave peak in the later stage of thermal cycling. In general, when the TGO amplitude is large, the thermal mismatch stress accumulated due to interfacial geometric discontinuity gradually dominates in the later stage of cycling and exceeds the stress-relief effect induced by plastic relaxation in the BC layer, thereby making the vicinity of the wave peak a more dangerous stress concentration region.

**Figure 9:**
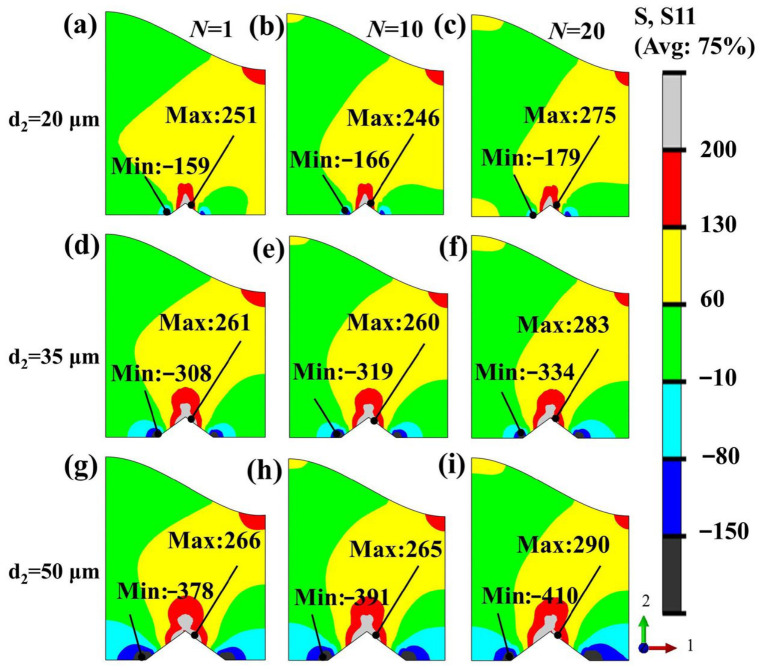
Distribution of radial stress (S11) on the BC layer surface under different grit sizes during thermal cycling: (**a**–**c**) d_2_ = 20 μm; (**d**–**f**) d_2_ = 35 μm; (**g**–**i**) d_2_ = 50 μm.

The above analysis focused on the effect of TGO amplitude on the stress evolution of the BC layer in the absence of grit inclusions. Considering that residual grit inclusions commonly exist at actual grit-blasted interfaces, the effect of grit size on the S11 stress distribution and its evolution in the BC layer was further investigated under the condition of A = 0.02 mm, as shown in Figure 9. As shown in Figure 9a–i, as the grit size increases from 20 μm to 50 μm during thermal cycling, the maximum tensile stress in the BC layer increases from 251 MPa to 290 MPa, while the minimum compressive stress decreases from −159 MPa to −410 MPa. This indicates that the introduction of grit particles alters the stress transfer path near the interface and causes more pronounced local stress concentration. Meanwhile, as thermal cycling continues, local stress concentration keeps accumulating under all conditions, suggesting that the thermal mismatch stress between the grit particle and the BC layer continues to increase during the later stage of cycling and exceeds the stress-relaxation effect caused by plastic deformation. In terms of stress distribution, tensile stress is mainly concentrated in the interfacial region above the grit particle, while compressive stress is concentrated near the two side tips of the grit particle, indicating that the region adjacent to the upper tip of the grit particle in the BC layer is more likely to become a critical site for crack propagation.

**Figure 10:**
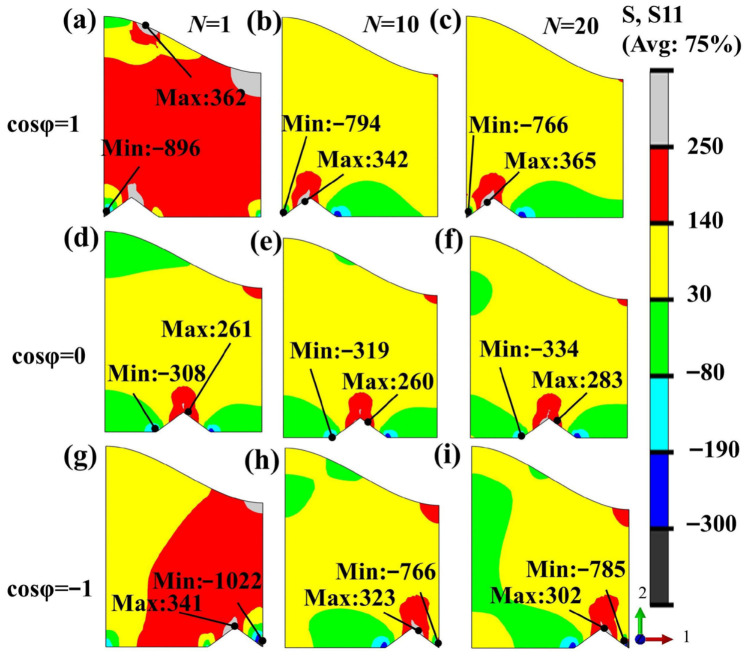
Distribution of radial stress (S11) on the BC layer surface under different grit positions during thermal cycling: (**a**–**c**) cosφ = 1; (**d**–**f**) cosφ = 0; (**g**–**i**) cosφ = −1.

A change in grit position may further alter the local stress transfer path and the distribution of critical stress regions. Therefore, under fixed conditions of TGO amplitude A = 0.02 mm and grit size d_2_ = 35 μm, the effect of grit position was analyzed. As shown in Figure 10, under fixed TGO amplitude and grit size, grit position significantly affects the S11 stress distribution in the BC layer. When the grit particle is located beneath the TGO peak, the stress concentration in the BC layer is the most pronounced; when it is located beneath the valley, the stress concentration is secondary; and when it is located in the mid-wave region, the stress level is the lowest. Specifically, the maximum S11 reaches 362 MPa when the grit is located beneath the peak, which is markedly higher than the 261 MPa obtained for the mid-wave condition. Under the valley condition, the maximum value in the initial cycle is 341 MPa, which lies between the other two cases. This indicates that the positional relationship between the grit particle and the undulated TGO interface significantly changes the local stress transfer path, resulting in obvious differences in the location and intensity of the critical stress region. Further analysis of the stress evolution during thermal cycling shows that when the grit particle is located beneath the valley, the maximum S11 in the BC layer decreases from 341 MPa to 302 MPa with increasing thermal cycles, showing a relatively obvious downward trend. In contrast, under the peak and mid-wave conditions, the stress peak changes little and even shows some rebound at the later stage. This result indicates that the region adjacent to the grit particle beneath the valley is more readily affected by stress relief induced by plastic deformation in the BC layer.

### 3.2. Crack Propagation Analysis Inside the BC Layer Under Single-Factor Conditions

Stress analysis can only be used to identify critical stress-concentration regions and general crack propagation tendencies, but it is difficult to quantitatively assess the specific influence of each factor on the crack propagation length inside the BC layer. To more intuitively reveal the effect of different factors on crack propagation inside the BC layer, an initial vertical crack with a length of 0.01 mm was pre-induced near the upper side of the grit tip in the BC layer (Figure 1b), and the crack propagation lengths under different conditions were further extracted and compared.

**Figure 11:**
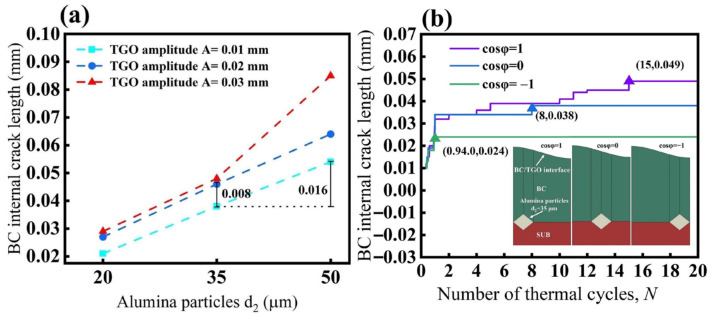
Crack propagation length inside the BC layer under the influence of different factors: (**a**) TGO amplitude and grit size; (**b**) grit position.

First, the effects of TGO amplitude and grit size on crack propagation length were analyzed, as shown in Figure 11a. With the grit position fixed at cosφ = 0, when d_2_ = 20 μm, the crack length increases only slightly as the amplitude increases from 0.01 mm to 0.03 mm, indicating that under small-grit conditions, the undulation of the TGO provides only a limited increase in the driving force for crack propagation. When d_2_ = 35 μm, the crack length further increases with increasing amplitude, and when the amplitude increases from 0.01 mm to 0.02 mm, the crack length increment is about 0.008 mm. In contrast, under the condition A = 0.01 mm, when the grit size increases from d_2_ = 35 μm to 50 μm, the crack length increases most markedly with TGO amplitude and exhibits a steeper growth trend in the large-amplitude range. These results indicate that, compared with TGO amplitude, an increase in grit size plays a more significant role in promoting crack propagation.

As shown in Figure 11b, under the fixed conditions of TGO amplitude A = 0.01 mm and grit size d_2_ = 35 μm, grit position also has a significant effect on the crack propagation length inside the BC layer. When the grit particle is located beneath the TGO peak, crack propagation is the most pronounced, and the final crack length reaches 0.049 mm, tending to stabilize after about the 15th thermal cycle. When the grit particle is located at the mid-wave position, the final crack length is about 0.038 mm and becomes essentially stable after about the 8th cycle. In contrast, when the grit particle is located beneath the wave valley, crack propagation is the weakest, and the final crack length is only 0.024 mm, becoming almost stagnant after the first cycle. These results indicate that when the grit particle is located beneath the peak, the stress concentration at the grit tip is more strongly superimposed with the tensile stress near the TGO geometric protrusion, thereby generating a stronger local driving force for crack propagation and leading to a longer crack length and longer growth duration. In contrast, when the grit particle is located beneath the valley or in the mid-wave region, local stress concentration is weakened, and crack propagation is significantly suppressed. Overall, the influence trends of these three factors on the crack propagation length inside the BC layer are generally consistent with the foregoing stress distribution analysis.

### 3.3. Coupling Effects of TGO Amplitude and Grit Size/Position on Crack Propagation Inside the BC Layer

In actual service of TBCs, crack propagation is not governed solely by a single factor such as grit particles. On the one hand, the TGO continuously thickens during cyclic oxidation and is accompanied by increasing interfacial undulation, and the resulting morphological fluctuation further intensifies the stress gradient near the interface. On the other hand, the size of residual grit particles and their relative positions within the undulated interface alter the local geometric constraint and stress concentration mode, thereby coupling with the evolution of TGO morphology. Therefore, to quantitatively characterize crack propagation behavior and identify the dominant factors and their interaction effects, a multi-factor coupled analysis involving TGO amplitude as well as grit size and position was further introduced in this study. The TGO amplitude (*A*), grit size (*B*), and grit position (*C*) of the thermal barrier coating were taken as variables. The Box–Behnken design (BBD)-based response surface method (RSM) was used to investigate their effects on crack growth inside the BC layer during thermal cycling. The design matrix is listed in Table 3, and a total of 17 models with different parameter combinations were constructed.

Based on polynomial fitting of the BC crack-propagation data, a quadratic regression model describing the relationship between the BC crack propagation length *L_BC_* and the three factors was obtained. Here, *A*, *B*, and *C* denote the TGO amplitude, grit size, and grit position, respectively. The regression equation is expressed in terms of actual factors and is applicable within the investigated design space of the Box–Behnken design.


(8) $$\begin{array}{cc} L_{BC}\;= & 0.016375-0.316667A+0.025B-0.031875C\\ & +38.3333AB+0.375AC+1.2BC\\ & -10A^{2}+10B^{2}-0.00575C^{2} \end{array}$$

Analysis of variance (ANOVA) of the regression model gave an overall model *p* < 0.0001, indicating that the established quadratic polynomial model can effectively reflect the statistical relationship between the factors and the response variable. The coefficient of determination was R^2^ = 0.9868, the adjusted coefficient of determination was 0.9698, and the predicted coefficient of determination was 0.7884. Since the difference between the latter two values was less than 0.2, the model shows good fitting ability and acceptable predictive performance. These statistical results indicate that the fitted model shows good agreement with the numerical results and acceptable predictive capability within the investigated parameter range.

**Figure 12:**
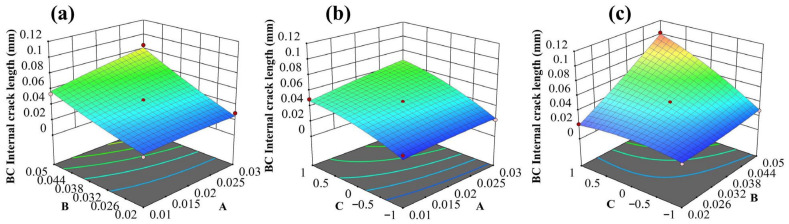
Response surface plots of BC crack propagation length under the interaction of different factors: (**a**) TGO amplitude and grit size; (**b**) TGO amplitude and grit position; (**c**) grit size and grit position. The color gradient represents the variation in BC crack propagation length.

On this basis, three-dimensional response surfaces and contour plots were generated from the quadratic regression model, as shown in Figure 12a–c. By comparing the slope changes of the response surfaces, the density of contour lines, and the curvature characteristics, the relative strength of main effects and the significance of interaction effects were analyzed. As shown in Figure 12a, under a fixed grit position, the crack propagation length increases with increasing TGO amplitude and grit size. Moreover, under larger grit-size conditions, the response surface exhibits a more pronounced slope in the TGO-amplitude direction, indicating that larger grit particles enhance the promoting effect of TGO amplitude on crack propagation, i.e., a certain synergistic effect exists between the two. Figure 12b reflects the coupling effect of TGO amplitude and grit position. The overall variation in the response surface is relatively gentle, and the contour lines are comparatively regular, indicating that the interaction between TGO amplitude and grit position is weak. Figure 12c shows that the interaction between grit size and grit position is stronger. The response surface exhibits substantial slope variation in both the grit-size and grit-position directions, and the contour lines show obvious bending and inclination, indicating that the effect of grit size on crack propagation changes significantly with its position. Overall, the coupling effect of grit size and grit position exerts the most significant promoting influence on crack propagation inside the BC layer.

**Figure 13:**
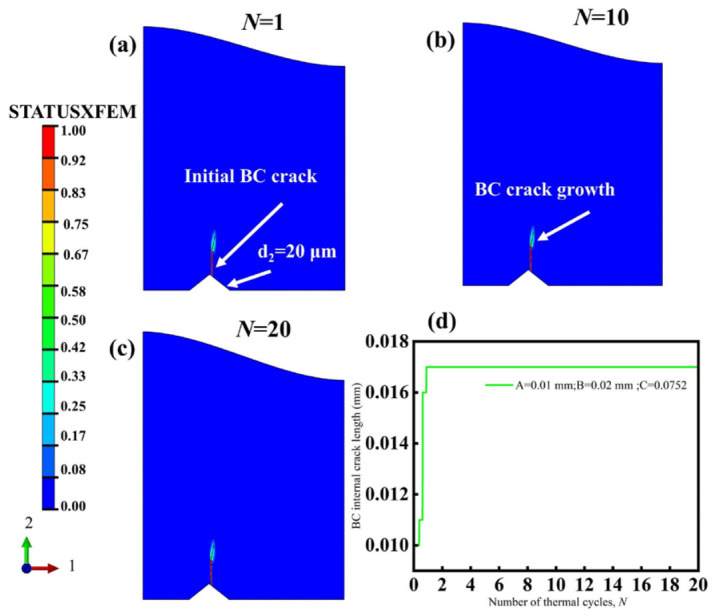
Finite element validation results for crack propagation inside the BC layer under the optimal parameter combination obtained by response surface optimization: (**a**–**c**) crack propagation morphologies; (**d**) crack-length evolution curve.

To further verify the predictive accuracy of the regression model, the minimum crack propagation length inside the BC layer was taken as the optimization objective, and the optimal parameter combination of the response surface was obtained as *A* = 0.01 mm, *B* = 0.02 mm, and *C* = 0.0752. Physically, this optimal parameter combination corresponds to a reduced local stress-concentration state, in which the combined effects of a smaller TGO amplitude, smaller grit size, and a favorable grit position suppress the propagation tendency of the crack in the BC layer. Substituting these parameters into the regression equation yields a predicted crack propagation length of 0.019 mm inside the BC layer. A further finite element model was then established based on this optimal parameter combination, and the crack propagation result is shown in Figure 13. As seen from Figure 13a–c, under this condition, the pre-induced crack on the BC surface experiences only slight propagation during the initial stage of thermal cycling and then remains nearly stable. No obvious crack deflection or rapid extension occurs during the later stage of thermal cycling. Figure 13d shows that the crack length increases from the initial 0.01 mm to about 0.017 mm after the first thermal cycle and then hardly increases with further cycling. This indicates that, under the optimized parameter combination, the local stress concentration induced by the combined action of TGO amplitude, grit size, and grit position is effectively suppressed, and the driving force for crack propagation is significantly reduced. The FE comparison provides supportive numerical verification of the regression model within the investigated parameter range, rather than independent experimental validation.

## 4. Conclusions

A two-dimensional thermo-mechanical coupled finite element model of a thermal barrier coating containing residual alumina grit inclusions and an undulated TGO interface was established. By combining the UEXPAN subroutine, XFEM, and response surface methodology, the effects of TGO amplitude, grit size, and grit position on stress evolution and crack propagation in the BC layer during thermal cycling were systematically investigated. The main conclusions are as follows:

1. Residual grit particles significantly modify the local stress distribution in the BC layer and promote crack propagation. Tensile stress is mainly concentrated in the region above the grit particle, making this area the critical site for crack development. Larger grit particles and unfavorable grit locations further intensify local stress concentration and increase crack propagation in the BC layer.
2. Although TGO amplitude affects the stress evolution in the BC layer, the crack propagation behavior is more strongly governed by grit-related factors. Among the three variables considered, the influence of grit size is the greatest, followed by grit position, while the effect of TGO amplitude is relatively weaker.
3. Response surface analysis shows that the interaction between grit size and grit position is the most significant among all factor combinations. A quadratic regression model for BC crack length was established based on the Box–Behnken design, and the optimized parameter combination for minimizing BC crack propagation was successfully obtained. The predicted result agrees reasonably well with the FE verification, indicating that the regression model provides supportive numerical verification within the investigated parameter range.

## Figures and Tables

**Table 1:** Plastic parameters of bond coat (MCrAlY) [28].

Plastic Parameters	Temperature/(°C)									
	400		600		800		900		1000	
σ/(Mpa)	1100	2500	1100	2200	300	380	45	60	10	15
ɛ_p_	0	0.23	0	0.30	0	0.02	0	0.02	0	0.01

**Table 2:** Thermal–mechanical properties of TBC.

Material	Temperature/ (°C)	Elastic Modulus/ (GPa)	μ	Density/ (kg∙m^−3^)	CTE/ 10^−6^∙°C^−1^
TC (8YSZ)	20	48	0.10	5280	9.0
	200	47	0.10	5280	9.2
	600	40	0.10	5280	10.1
	800	34	0.11	5280	10.8
	1100	22	0.12	5280	12.2
BC (MCrAlY)	20	200	0.30	8100	13.6
	200	190	0.30	8100	14.2
	600	160	0.31	8100	15.2
	800	145	0.32	8100	16.1
	1100	110	0.35	8100	17.6
TGO (α-Al_2_O_3_)	20	400	0.23	4000	8.0
	200	390	0.23	4000	8.2
	600	370	0.32	4000	8.7
	800	355	0.32	4000	9.0
	1100	320	0.33	4000	9.6
SUB (Inconel DZ125)	20	220	0.31	8200	14.8
	200	210	0.32	8200	15.2
	600	170	0.33	8200	16.2
	800	155	0.34	8200	16.9
	1100	120	0.35	8200	18.0
Alumina particle (Al_2_O_3_)	20	380	0.27	38,700	5.08
	220	369	0.27	38,700	5.90
	420	370	0.27	38,700	6.73
	620	355	0.27	38,700	7.55
	1020	320	0.27	38,700	9.20

**Table 3:** Response surface design matrix and results.

Number	*A*	*B*	*C*	BC Internal Crack Length (mm)
1	0.01	20	0	0.021
2	0.03	20	0	0.029
3	0.01	50	0	0.054
4	0.03	50	0	0.085
5	0.01	35	−1	0.024
6	0.03	35	−1	0.022
7	0.03	35	1	0.062
8	0.01	35	1	0.049
9	0.02	20	−1	0.019
10	0.02	50	−1	0.028
11	0.02	20	1	0.021
12	0.02	50	1	0.102
13	0.02	35	0	0.046
14	0.02	35	0	0.046
15	0.02	35	0	0.046
16	0.02	35	0	0.046
17	0.02	35	0	0.046

## Data Availability

The original contributions presented in this study are included in the article. Further inquiries can be directed to the corresponding authors.
